# Atlanta Academy of Medicine

**Published:** 1874-02

**Authors:** James B. Baird

**Affiliations:** Reporter


### Atlanta Academy of Medicine.

JAMES B. BAIRD, M.D., Reporter.

Dr. John G. Westmoreland, First Vice-President, presiding.

Dr. V. H. Taliaferro presented the following report, sent to the academy through him, by Dr. A. K. Smith, of the United States Army.

Santa Fe, New Mexico, July 22, 1873.

I was called, a few evenings since, to visit a Mexican woman in labor. The fact of being called forewarned me that it was a difficult case, as the Mexicans attend to these little things themselves, and very rarely call in a medical man. I found my patient had been in labor twenty-four hours, and that the nature of the case had baffled the skill of the wise women who usually assist on such occasions. Upon making my examination I found both arms of the child in the vagina, the chest presenting, and the uterus so firmly contracting that it was impossible to pass even a finger up to find a foot or knee by which I could turn. I placed her in the breast and knee position, but succeeded no better in my manipulations, and as the pulseless cord, which protruded, also, into the vagina, showed conclusively that the child was dead, I resolved to eviscerate and bring the child away piecemeal. Here was a case which I never saw before, the chest presenting and the head and legs doubled over the back. At the same time the womb was contracting violently and with scarcely any intermission. The child being dead I considered myself justified in resorting to any measure to save the mother, and I accordingly went to my hospital, a few rods distant, for my instruments and chloroform. I returned with many misgivings as to the result, for it was an exceedingly bad case. But imagine my surprise and great delight when I found the child with placenta and membranes lying on the floor. During my absence a most violent pain had come on, spontaneous evolution had occurred, and the child had descended by the breech its legs doubled over its back, and its head crushed like an egg-shell.

The mother has recovered without a single bad symptom, her only medication being a full dose of opium, given shortly after the birth of the child.

Dr. Battey considered the case unique; thinks the thighs must have been dislocated. Spontaneous version, in his opinion, is not so rare as generally thought; it has occurred more than once in his practice; thinks change in the *position* of the vertex common, and that the failure on the part of physicians in discovering this change is due to want of care in their first examination; he believes that the position of the child is continually changing before pains come on; he cannot say that podalic version often occurs. In two cases of his own the shoulder has presented at the os, half an hour later the breach presented and the child was expelled that way without turning,

Dr. Dupre was called in consultation to a woman who had been in labor for twelve or fourteen hours, arm presented; found it impossible to pass the hand into the uterus from the violent contractions; chloroform, tartar emetic and venesection had been resorted to; in a short time spontaneous version occurred; believes that the force of the hand applied against the body of the child started or favored the change in position. A similar case has been reported to him by a medical friend, in which the presentation was transverse; thinks the profession is at fault in neglecting, to the extent that it does, external manipulation; in difficult labor is sure that he has facilitated parturition by steady pressure on the fundus of the uterus, synchronously with the expulsive pains.

Dr. Battey desired to impress the importance of an early diagnosis both of the presentation and the position; he believes that in many cases difficulties may be warded off and a malposition corrected when recognized early. In one or two instances, by mistaking the position of the child, has attempted to change the position contrary to physiological laws; fortunately he was foiled in his efforts, and nature did the work; thinks that for the accoucheur to assist nature it is necessary for him to have a correct idea of the position of the child in utero.

Dr. Taliaferro disagreed with Dr. Battey as to the frequent change of position; also as to the facility with which changes could be brought about by external palpation.

Dr. Battey explained that distinction is to be drawn between position and presentation.

Dr. E. A. Flewellen reported the case of a primapara, æt. thirty. In labor. Examined per vaginam, and felt, as he thought, membranes presenting at the os. The head of the child was presenting. There was no relaxation of the external parts; was tempted to rupture the membranes, but concluded to wait a few hours. At the end of several hours there was no change. Membranes seemed to be very thin. He could not feel, upon a thorough examination, the mouth of the womb around the membranes. Passing finger up towards the promontory of sacrum, found the os about the size of a nickel, high up in the hollow of the sacrum. He then discovered the presenting part to be the anterior wall of the uterus pressed upon by the head of the child instead of the membranes. He proceeded to bring the os down, and gradually dilated it by mechanical means; delivery was then easy.

Dr. Rauschenberg had met with a few similar cases in which the pressure of the head upon the anterior wall of the uterus gave the sensation to the finger as if the membranes were presenting; considers it due to neglect in the early stages of labor; when the os is high up it should be brought forward so that the axis of the uterus will correspond to the inlet of the pelvis; thinks that external manipulation is almost always to be condemned; can only do damage by pressure; gentle frictions will excite all the contractions the womb chooses to make; may change the position by pressure upon the child's head directly; when he wishes to change the presentation he always trusts to getting his hand into the uterus.

Dr. Battey was surprised at the expressions of Dr. Rauschenberg when he considers that external manipulation for diagnosis is one of the crowning achievements of modern German obstetrics; suggested that in cases where there was bulging of the anterior wall of the uterus, as in the case just reported, that there might be some local disease weakening the wall of the uterus at that point. Adjourned.

———

DECEMBER 15, 1873.

Dr. Ch. Rauschenberg, Second Vice-President, in the chair.

Dr. S. H. Stout called the attention of the Academy to the damage frequently done in the use of the *nasal douche,* by water passing along the Eustachian tube and causing inflammation of the middle ear. Has seen injurious effects in his own practice, and has heard its use invariably condemned by several prominent physicians with whom he has talked on the subject.

Drs. Dupre, McDowel, J. G. Westmoreland and others agreed with Dr. Stout, and those who had used it had abandoned it on that account.

Dr. Anderson reported an epidemic of a peculiar type of fever in regard to which he wished to get the opinion of the Acadamy. The fever lasts from three to five days—severe tonsilitis laryngitis; thought one case was true diphtheria. No pain in back or limbs. *Treatment:* tonics with caustics and astringents to throat. The opinion was expressed by several of the members that it was a form of catarrhal fever, or simple influenza.

Dr. Battey: A child three years of age, there is a history of a diarrhœa four months ago, which continued one month, and was checked; and for the past three months there has been anasarca. No evidence of fluid in the cavities, and can detect no organic lesion to account for the persistent anasarca. Has been treated by diuretics, etc. Asks the opinion of the Academy on the case.

Dr. Stout asked if opiates had been used extensively for the diarrhœa.—Answer, yes.—It very frequently occurs in the prolonged use of opium in such cases you have a disposition to anasarca even in adults. The water has a tendency to effuse through the weakened capillaries, and often opiates are given to check the diarrhœa when tonics are needed. Suggests tonics, vegetable astringents, etc.

Dr. Battey agrees with Dr. Stout in his views—the exceeding persistency of the dropsical condition, is the point to which he wished to call particular attention. Does not remember ever to have seen a case of so long continuance without organic trouble. Child is anæmic and slightly purpuric spots on extremities. Digestive organs in apparently good condition; appetite good; rather too good; bowels a little constipated.

Dr. Taliaferro asked if there were any history of tubercle or scrofula in the family?

Dr. Battey thinks possibly there may be. In a case reported in the JOURNAL, of typho-malarial fever in a boy thirteen years old, after the subsidence of the fever, he found him, one night, breathing badly, pulse 45; effusion in the chest; tenderness in the region of kidneys, and on following morning albumen found in the urine.

Under a treatment of iron and digitalis there was a prompt and rapid recovery from this condition. Quite possible there may be some renal trouble in this case.

Dr. Flewellen, by request, reported the following case, which came under his notice some years ago: A negro woman, house servant, had borne two or three children, and supposed to be pregnant again. She remained in this condition several years, in good health and attending to her business as usual, when she suddenly dropped dead. Upon a post-mortem being made, a portion of an inferior maxillary bone, a canine tooth, and a fragment of a skull about the size of the hull of a black walnut were found in the tumor with some hair, a portion of which was attached to the skull, the rest loose in the sac.

Dr. Battey was reminded by this case of a case of ovariotomy, reported some years ago. Within the tumor were a quantity of adipose substance very much like "cotton-seed butter," a number of teeth and a portion of bone bearing resemblance to petrous portion of temporal bone. Some of the teeth were adherent, while others were loose in the sac. There was one large canine tooth and a number of small teeth, some of which were perfect, others not. He believed this to be a dermoid cyst of the ovary, not an extra-uterine pregnancy.

Dr. W. F. Westmoreland asked if Dr. B. did not consider this a case of extra-uterine pregnancy, how he accounted for those teeth, bone, etc., which were to him evidently the remains of a fœtus?

Dr. Battey contended that there was no impregnation. The tumor had existed for about eighteen years—was seen when she was about seventeen years of age, before marriage, and was then quite small; thinks she had one living child and had one abortion. The tumor was tapped during the war, and a quantity of buttermilk-looking substance withdrawn. Believes the fact of an adult canine tooth being in the sac precludes the idea of pregnancy. Similar cases have been reported in the case of very young girls with teeth, hair, etc., in the sac. Believes this tumor was originally a composite cyst. Weight of the sac and contents, thirty pounds. A small body, possibly the ovary on that side, was attached to under side of tumor; thinks it originated from a grafian vesicle. Fallopian tube entirely separate from the tumor. Other ovary healthy. General health now perfect.

Dr. W. F. Westmoreland: Can't see how we can have teeth or hair, etc., in an ovarian cyst, without pregnancy. There was either an impregnation of the child or mother. Clear to him that a grafian vesicle was impregnated. In case reported by Dr.

F., you have progression, and in other cases you have the death of the fœtus and its discharge through the walls of the abdomen, by the rectum, or in some other way. Can't conceive of such a case as that reported by Dr. B. without impregnation.

Dr. Battey: Dr. W. does not account for the fact that there were both adult and fœtal teeth, nor for the disappearance of the fœtus and its skeleton. In cases of extra-uterine fœtation, we have a well developed fœtus, which after years may become mummified, or there is a discharge of bones, etc., which are discharged as the *skeleton* of a fœtus. In the case reported there was no abscess and no discharge of bone, etc. Believes this was not a case of extra-uterine pregnancy, but a mere aberration of the procreative faculty. A graafian vesicle may take on a heterologous growth, just as may be seen in glands or the changes in arteries, etc., which may account for these foreign substances found in an ovarian cyst.

Dr. W. F. Westmoreland: Dr. B. believes that in cases of ventral pregnancy we usually have an abscess and a discharge of the contents of the tumor, but there are a number of cases in which we have no abscess. As to the adult tooth, the tumor was 18 years in growing, and in that time we may have an adult tooth. The bones of the fœtus were absorbed, but the teeth are not so readily absorbed, and may remain for years in the sack. No difficulty in accounting for the cotton-seed butter, often have such a deposit. Believes it to be a true ventral pregnancy; can't conceive of an ovum developing into a fœtus in one case and not in another.

Dr. Rauschenberg cannot comprehend how teeth or bones, distinctly representing in shape parts of the human organism, as Dr. Battey has stated, could occur in a tumor of that kind, or anywhere else in the body of a female, without previous impregnation of an ovum. If the formations of which Dr. Battey speaks were, histologically considered, bone and teeth; if they exhibited under the microscope the anatomical structure of these tissues, their existence must have been the result of the development of an impregnated ovum, and if the balance of the skeleton was not present, there must have been either incomplete development or degeneration and resorption afterwards, or both. Any other interpretation of their genesis is entirely inadmissible under the present laws of physiology.

Dr. Battey will exhibit the bones and teeth—except the canine tooth, which is not in his possession—to the Academy. Agrees with Dr. R., that can't have formation of bone and teeth contrary to physiological laws, and will attempt to account for them according to the laws of pathology. We have scirrhus tumor in the mammary gland, and under a similar law may have formation of teeth and bones in an ovarian tumor. Dr. Nottingham reported such a case to the Georgia Medical Association.

Dr. R.: The occurrence of cancerous degeneration of the mammary gland bears no analogy whatever to the formation of bones and teeth in the cysts under consideration. In the pathological structure of the former the component normal elements of the mammary gland can be distinctly recognized, and the abnormity consists in the change in size and shape of the cells of a certain part of the tissue in relation to that of another. When teeth and bone, distinctly shaped and recognizable as certain parts of the human frame, are found anywhere about the ovaries or sexual organs of a woman—and they certainly will never be found anywhere else imbedded in the tissues of a female—we are dealing with an imperfectly developed, or the remnants of a degenerated fœtus, always the product of the impregnation of an ovum. Physiology admits no other explanation.

Dr. Battey signifies his intention of presenting, at some future time, an essay on this subject. He was requested to present a portion of the bone and teeth to the Histological and Pathological Society for microscopical investigation.

---

December 22, 1873.

Dr. John G. Westmoreland, First Vice President, presiding.

Dr. J. G. Westmoreland reported a case of uterine cancer. The body of the uterus is considerably enlarged, so that the neck projects at the vulva; the cervix is ulcerated; the patient suffers intensely from laminating pains; has been able to procure no relief except only temporarily from the free use of opium; there is do hope of effecting a cure, but would like to hear suggestions for controlling the pain. Several members responded, and a trial of electricity, iodoform in solution with ether, gum opium, chloral, and the hypodermic use of morphine were suggested.

Dr. J. T. Johnson has never seen any permanent relief from electricity; its effects in his hands are only momentary; he urged the hypodermic use of morphine, as in a case necessarily fatal there need be no objection to its free and protracted administration.

Dr. Westmoreland admitted that the effect of morphine was more prompt when given hypodermically, but thinks it is more transient. Adjourned.

---

DECEMBER 29, 1873.

Dr. W. S. Armstrong, Second Vice-President, presiding.

Dr. Battey reported a case which he had under treatment, eighteen months ago; a married lady suffering from an old stricture of the cervix uteri. The result, by no means rare, of active treatment by caustic for cervical endometritis several years before. The menses escaped guttatim and required four weeks for their discharge. Treatment by dilatation and iodine: this is always his treatment; never uses uterotome. This treatment was followed by improvement in menstruation which became regular, occurring at the usual period, and observing the usual intervals of rest. She mentioned in May, during treatment, that six weeks had passed since her last menstrual flow. He suggested pregnancy, but she denied the probability of such a cause, her youngest child being fourteen years old, and she near the "turn of life." He was disposed to agree with her, but thinks it a good rule, and one that should invariably be applied, that when a married woman, living with her husband, misses her courses, pregnancy should be regarded as the cause until the negative is proven. The negative view in this case was strengthened by the fact that prolonged cervical or corporeal endo-metritis is productive of barrenness. The return of the menses and their regular continuance, on the other hand, with the nausea and vomiting, tended to confirm the suspicion. During the entire summer and fall the case remained an enigma to him; there was a slight enlargement of the uterus, with a peculiar discharge. One night, early in January, he was called to see her and found her suffering great pain. He made a vaginal examination, and his finger came in contact with a rough body protruding from the os. It was removed with forceps and proved to be a fœtal bone. The os was dilated, and an entire skeleton, which he presented for inspection, removed. There were no membranes, the bones being as clean as they now are. The offensiveness of the menstrual discharges was not greater than is observed in some cases of endo-metritis. He judged that the bones belonged to a three months' fœtus.

Dr. Stout thought that this case confirmed his views in reference to retained placenta. Has always deprecated the practice of rapidly and forcibly dilating the os after abortion, for the purpose of removing the placenta. He advocated the practice of non-interference, and argued that it should be left to nature to deliver the placenta. Once saw a woman die from attempts to remove a placenta, after an abortion at the fourth month. Thinks it safer to risk the occurrence of septicæmia than to forcibly remove the placenta.

Dr. Battey thought the proper course to pursue is, immediately after the expulsion of the fœtus, while the os is dilated—assuming that nature has not at once cast off the little placenta—to pass the finger into the uterus, and placing it upon the placenta, exercise gentle traction, and at the same time a rotary motion, assisted by conjoined manipulation. But after delay, advises reliance upon nature, and condemns the attempt to prize open the uterus. He once had a patient to die from retained placenta, under the following circumstances: She had recovered from an abortion, and been up and about for a week. He was hurriedly summoned to her for an alarming hemorrhage, which occurred suddenly, causing her to faint while sitting at the tea-table. Found her pulseless and breathing with difficulty. Tamponed the vagina. In two hours reaction had taken place, so that the pulse could be felt at the wrist. She was subsequently attacked with tetanus. The discharges from the uterus became offensive, and she died of uterine tetanus.

Dr. Stout explained that he did not refer to cases seen at the time abortion occurred.

Dr. Dupree, a number of years ago, met with the case of a negro woman who miscarried near full time, the result of a rough ride in a wagon. Four or five weeks afterward he was called to see her, and found that she had tetanus. She died forty-eight hours later. The delivery was represented to have been complete. The uterus was entirely normal. What could have been the cause of the tetanus?

Dr. Battey said that the books speak of "puerperal tetanus." Thinks it an error to designate such cases as puerperal, and thinks the term uterine tetanus should be substituted. Will refer to two cases illustrating this position: First, the case of Dr. Simons, of Charleston—obliteration of the uterine cavity—suppression of the menstrual discharges—nothing puerperal, but great uterine irritation. Second, a case of his own—a lady about the climacteric period. Has endo-metritis. Has been under his notice for several years. No local treatment, to which she is very averse. Hopes and expects that the change of life will effect a cure. She has had for several years a stiffness, spasm and pain of the masseter muscles. This condition lasts for a week, ten days or two weeks; gradually increasing until the jaws become locked. There are no general convulsive movements. She has had a dozen of these attacks. Has seen her in half of them. Thinks the nerves supplying the inner surface of the womb are impressed, just as are the nerves surrounding a punctured wound. Uterine tetanus is as inexplicable as cases of traumatic tetanus.

Dr. Taliaferro felt obliged to dissent from the views expressed by Drs. Battey and Stout in reference to the management of the retained placenta and dead fœtus. No necessity exists for forcible dilatation, it is dangerous—but foreign bodies in the cavity of the uterus should be removed, especially if they are putrid. The os may be slowly and safely dilated by means of tents gradually increased in size. The cervix dilates from contact with the foreign substance; it is not even necessary for the tent to press upon the tissues. Barnes' dilator can be substituted for the tent if a freer dilatation should be necessary.

Dr. Stout thought that a tent at that stage was liable to produce inflammation. It acts as a plug and retains the blood, etc., in the cavity of the womb. The uterus is in a state of exalted irritability. He has, however, never tried the tent.

Dr. Taliaferro: The irritation from the tent is less than from the retained placenta; the cervix can bear, without inconvenience, a greater amount of irritation than the body of the uterus. He expects no danger from a tent of an unirritating kind—the cloth or sea-tangle. Does not use the sponge tent. The cloth tent drains the uterus, especially if saturated with glycerine.

Dr. Rauschenberg has used the sponge tent to control hemorrhage in two cases of retained placenta, after abortion, recently. Introduced the tent into the cervix and tamponed the vagina, at the same time applying ice-water to the abdomen. In both cases he found the tent and placenta in the vagina next morning. Thinks there is no danger in this mode of treatment, and in the majority of cases it will prove successful.

Dr. Love agrees with Dr. R. Was called to see a woman who had aborted—found the cord protruding at the os. Introduced a sponge tent enveloped in India-rubber and tamponed the vagina; this tampon being saturated with glycerine. Ergot per orem; sixteen hours later removed the tampon and found the placenta in the vagina. Cellulitis and metritis followed; a pelvic abscess formed, which opened into the vagina. He reported a case some time ago in which post partum hemorrhage was successfully treated by electricity. Found the woman bleeding freely, pulseless and almost breathless. Placenta detached, but no contraction. He introduced his hand into the cavity of the uterus, but failed to cause contractions. He then applied one pole of a battery enveloped in a wet towel to the spine of the woman, the other to his own elbow, his hand remaining in the uterus; immediate and firm contraction ensued. He determined to apply this treatment in cases of abortion, and force the contractions of the uterus to expel the placenta.

Dr. Battey stated that he had no personal experience in this use of tents. The practice, however, does not commend itself to his judgment when he considers that formidable metritis and metro-peritonitis has resulted from the use of the sponge tent in the unimpregnated uterus. To be effective the tent must pass the internal os.

Dr. Baird inquired if Dr. Battey was not afraid to risk leaving his patients with retained placenta after abortion, without the precaution of tamponing?

Dr. Battey replied, that he did not consider it unsafe if the patient was confined to bed and ergot actively administered. If he should feel any solicitude, regards the tampon safer than the tent. Adjourned.

